# Co-Evaluation of Lactate, Base Excess, and Albumin as Predictors of Mortality in Sepsis by Excluding the Factors That Affect Their Levels: An Observational Study †

**DOI:** 10.3390/biomedicines13081932

**Published:** 2025-08-08

**Authors:** Aysenur Gur, Elif Ozturk Ince, Nalan Metin Aksu

**Affiliations:** 1 Etimesgut Şehit Sait Ertürk State Hospital, Etimesgut, Ankara 06790, Turkey; 2 Department of Emergency Medicine, Hacettepe University, Sıhhiye, Ankara 06230, Turkey; elifozturk@hacettepe.edu.tr (E.O.I.); nametaksu@yahoo.com.tr (N.M.A.)

**Keywords:** sepsis, septic shock, lactate, albumin, base excess

## Abstract

**Purpose:** Our aim was to determine the benefits of serum lactate, albumin, and base excess (BE) values in predicting prognosis and mortality in sepsis when evaluated together. **Methods:** We performed a prospective observational study. We included 217 patients who were 18 years old or older, admitted to the Adult Emergency Department at Hacettepe University Hospital between May 2019 and July 2020, and who had two or more SOFA scores. We evaluated admission hour, 24th hour and 48th hour lactate, albumin, and BE. **Results:** A decrease in 0, 24th, and 48th hour albumin values increases the mortality of sepsis patients. An increase in the 24th hour lactate value increases hospital mortality. Changes in BE values had no effect on hospital mortality. Hospital mortality increases as 24 h lactate clearance decreases. Alactic base excess has no effect on mortality. The AUC values of lactate and albumin are significant, but their sensitivities are low. The AUC value for 24 h lactate clearance is significant, but the sensitivity of the AUC value is low. **Conclusions:** Contrary to the literature, lactate, albumin, and BE were found to have low sensitivity in determining prognosis and mortality. When factors that may influence serum lactate, albumin, and base excess (BE) are excluded, the values of these biomarkers decrease when predicting mortality.

## 1. Introduction

Sepsis patients constitute a proportion of critically ill patients admitted to emergency departments (EDs). Sepsis is the leading cause of death from infection and is a medical emergency [1]. The emergency management of sepsis includes early diagnosis, hemodynamic resuscitation, source control, and appropriate antibiotic administration [2]. It is possible to change the morbidity and mortality results due to sepsis thanks to appropriate ED management.

Sepsis is currently defined as life-threatening organ dysfunction caused by an impaired host response to infection; that is, patients with a sepsis-related (sequential) organ failure assessment (SOFA) score of 2 or higher in patients with suspected infection are defined as septic. Septic shock is the need for a vasopressor to maintain mean arterial blood pressure ≥ 65 mmHg despite adequate fluid resuscitation and a serum lactate level > 2 mmol/L [1].

The SOFA score consists of six criteria affecting organ systems (respiratory, cardiovascular, renal, neurological, hepatic, and hematological) that are usually measured at admission to the intensive care unit (ICU). In recent years, it has been used both in the evaluation of the response of critically ill patients to treatment and in the evaluation of acute morbidity [3].

Although biomarkers add accuracy and objectivity, their usefulness in predicting mortality alone is limited due to their low specificity and sensitivity. Recent studies have shown that the combined use of biomarkers and scoring systems is successful in predicting mortality [4,5]. Some studies indicate that the simultaneous use of multiple biomarkers is advantageous with or without a scoring system [6].

There are many more biomarkers for sepsis than for any other disease processes (more than 170 biomarkers). The wide variation in the number of biomarkers is probably associated with the intricate pathophysiology of sepsis, which encompasses numerous inflammatory mediators and additional pathophysiological pathways [6,7]. Lactate is a strong marker of disease severity in established sepsis; abnormal lactate levels are more likely to be the result of impaired tissue oxygen utilization [8]. Since albumin is a negative acute phase reactant, it can reflect the severity of inflammation. Given that hypoalbuminemia is a common finding in chronic disease, data from community-acquired sepsis studies have suggested that hypoalbuminemia is associated with infection and have shown that albumin may serve as an independent risk parameter. Base excess (BE) in sepsis indicates the kidneys’ inadequacy in maintaining acid–base balance in the metabolic acidotic environment caused by impaired tissue oxygen utilization [9,10].

Our aim in this study is to determine the benefits of serum lactate, albumin, and BE values by excluding the factors that affect their levels in predicting prognosis and mortality in patients with sepsis when evaluated together. Identifying biomarkers in critically ill patients that can be used to predict prognosis and mortality in the emergency department would enhance therapy management.

## 2. Materials and Methods

### 2.1. Study Design

This study was designed as a prospective observational study. Ethics committee approval (registration number GO 19/510) was obtained from the Hacettepe University Clinical Research Ethics Committee. Our study was conducted in accordance with the Declaration of Helsinki (as revised in 2013). Patients over the age of 18 who applied to the ED between May 2019 and July 2020, had signs of infection, had a SOFA score of 2 and above, and provided written informed consent from themselves or their relatives to participate in the study were included.

Patients’ gender, age, medical history, SOFA scores on admission, sources of infection, 0-, 24, and 48 h lactate, standard BE, and albumin values and outcomes were recorded.

Septic shock patients were recruited according to the definition in the latest sepsis guideline published by the Surviving Sepsis Campaign in 2016 [1]. Patients who required vasopressors to maintain mean arterial blood pressure ≥ 65 mmHg despite adequate fluid resuscitation and had a serum lactate level > 2 mmol/L were considered to be in septic shock.

Our hospital is a university hospital and provides training consistent with the current medical literature. The sepsis patients included in our study were treated in accordance with the current 2016 sepsis guidelines [1] and our hospital’s infection control policies. Our study is an observational study and we did not intervene in clinical decisions or treatment choices.

The SOFA score was calculated according to Table 1.

Lactate is expressed in mmol/L and albumin is expressed in g/dL.

The standard BE was calculated according to the formula BE = 0.02786 × pCO2 × 10^(pH − 6.1)^ + 13.77 × pH − 124.58 [10].

The alactic base excess (ABE) was calculated according to the formula ABE (mmol/L) = standard BE (mmol/L) + lactate (mmol/L) [11].

Lactate clearance (LC) was calculated as 24 h and 48 h clearance according to the formula LC(%) = (admission lactate − lactate at 24/48 h)/admission lactate × 100 [12].

Patients over the age of 18 with signs of infection and a SOFA score of 2 or higher were included. Informed consent was obtained from the patients included in the study. In our study, the follow-up period for each patient was 90 days.

The exclusion criteria were as follows:

- Lactate, BE, or albumin follow-ups were missing for any reason;
- Insufficient information about the outcome could not be found;
- Type 2 lactatemia reasons (using metformin, chronic liver disease, etc.);
- Having chronic kidney disease;
- Antibiotics not started;
- Having acute gastrointestinal bleeding;
- Pregnant women;
- Trauma patients;
- Acute myocardial infarction patients;
- Repeated admissions of the same patient.

**Figure 1:**
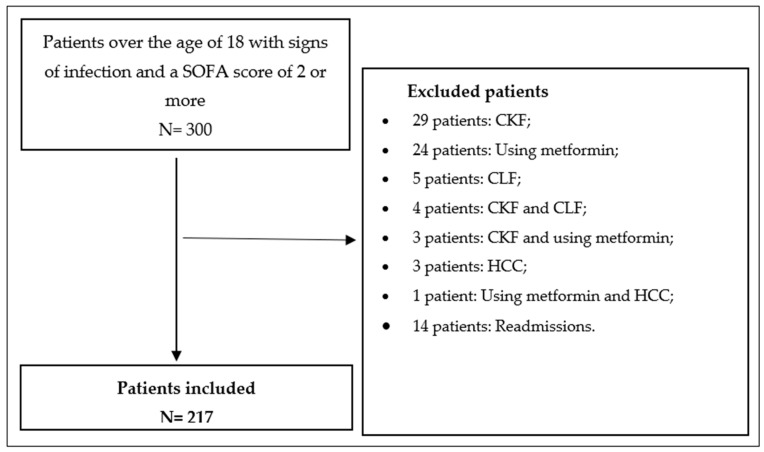
Study flow chart. *Abbreviations: CKF: chronic kidney failure, CLF: chronic liver failure, HCC: hepatocellular carcinoma*.

We excluded parameters that can affect serum lactate, albumin, and BE levels (see Figure 1).

### 2.2. Stastistical Analysis

Analyses were performed using Statistical Package for the Social Sciences (SPSS Statistics 23) and the R Shiny application easy receiver operating characteristic (easyROC). Frequencies and percentages are given for qualitative variables; means, standard deviations, medians, and minimum and maximum values are given for quantitative variables. The relationship between two qualitative variables was examined by chi-square analysis. As a result of the chi-square analysis, Fisher’s exact test was used when the proportion of cells with an expected frequency of less than 5 exceeded 25%, and Pearson chi-square test results were determined when it was below 25%. Examining the difference between the two independent groups in terms of quantitative variables, the assumption of normal distribution was checked first. While checking this assumption, a Kolmogorov–Smirnov test of the normality and histogram, a QQ plot and box–line graphs were used.

An “Independent Samples *T* Test” was used for the cases where the assumption of normal distribution was provided; in cases where it was not provided, it was applied with the “Mann–Whitney U Test”. Mean and standard deviation values are given for normally distributed variables; medians and quartiles are given for non-normally distributed variables. ROC analysis was performed to determine the cut-off value for mortality in terms of 0, 24, and 48 h lactate, BE, and albumin variables. In the ROC analysis, firstly, the area under the curve (AUC) and the significance of this area were examined. For the variables whose AUC was significant (*p* < 0.05), appropriate cut-off values were determined by looking at the sensitivity and selectivity values; *p* < 0.05 was accepted for the statistical significance level. Logistic regression analysis was performed in order to evaluate the statistically significant values together. In order to use lactate, albumin, and BE in predicting mortality, models were created by performing ROC analyses and logistic regression analyses. Lactate alone in Model-1, albumin alone in Model-2, and lactate and albumin together in Model-3 were evaluated. If ROC analyses and areas under the curve (AUC) were not found to be statistically significant, they were not included in the models. To construct the optimal combination of biomarkers, a logistic regression model was used for conversion. The model fit was assessed with a Hosmer–Lemeshow goodness-of-fit test and a correlation coefficient of r > 0.5. We considered a specificity of 85% to be significant for markers, and using a one-sided tail with a marginal error of 0.05 and a confidence interval of 95%, we calculated a sample of at least 176 [13].

## 3. Results

The total number of patients was 217. It was determined that 60.4% of the patients were male (*n* = 131). The mean age of the patients was 67.5 ± 16.5 years (the youngest was 19, the oldest 98). Of the patients included in the study, 90.3% (*n* = 196) had sepsis and 9.7% (*n* = 21) had septic shock. Pulmonary infections were found to be most frequently involved in the etiology of sepsis. Characteristics of the patients are given in Table 2.

Patients’ 0, 24, and 48 h lactate, LC, BE, ABE, and albumin values are analyzed in Table 3.

Of the patients, in 61.8% (*n* = 134), the infection source was found to be pulmonary infection. The second source was found to be urinary infections, at 11.5% (*n* = 25).

Of the total, 32.3% (*n* = 70) died in the hospital. Hospital mortality, according to lactate, BE, and albumin values of the patients, is analyzed in Table 4. A statistically significant relationship was found between albumin values and hospital mortality. Low albumin values increased hospital mortality. Additionally, the low lactate value at 24 h increased the hospital mortality. No statistically significant correlation was found between BE, 0, and 48 h lactate values and hospital mortality in sepsis patients. No statistically significant correlation was found between lactate, albumin, and BE values and hospital mortality in septic shock patients.

The effect of the SOFA score on mortality is given in Table 5. The SOFA score was found to have an effect on mortality in sepsis (*p* = 0.045), but it had no effect on mortality in septic shock (*p* = 0.188) in our study.

When mortality rates were analyzed according to ABE and LC in sepsis patients, a statistically significant difference was found only between 24 h LC and hospital mortality (*p* = 0.037), as shown in Table 6. Hospital mortality increased, while 24 h LC decreased. No significant correlation was found between ABE and LC and hospital mortality in patients with septic shock.

In order to use lactate, albumin, and BE in predicting mortality, models were created by performing ROC analyses and logistic regression analyses. Lactate alone in Model-1, albumin alone in Model-2, and lactate and albumin together in Model-3 were evaluated in Table 3. The AUCs were not found to be statistically significant in the ROC analyses for BE, ABE, and LC, as shown in Table 7.

When the initial lactate was evaluated alone, it was significant in Model-1 (*p* = 0.038), but the AUC was not significant (*p* = 0.707).

The initial albumin was significant in the model when evaluated alone (*p* < 0.001). Since the odds ratio (OR) was less than 1, the high albumin values provided protection. Low albumin values are a risk factor for mortality. The AUC was significant when albumin was used alone (*p* = 0.020); however, since the size of the AUC was below 0.70, its distinctiveness is not high.

When the admission lactate and albumin are used together, the AUC is significant (*p* = 0.019). However, since this model has the same AUC (0.604) as the model in which albumin is alone, it can be said that lactate does not contribute to the model and albumin can be used alone. Since the AUC is below 0.70, the sensitivity values are also low, as shown in Table 6.

No significant results were obtained for hospital mortality in the models made according to lactate and albumin values at the 24th hour.

The lactate + albumin model (Model-3) was found to be statistically significant (*p* = 0.013) in the modeling performed according to lactate and albumin values at the 48th hour. However, since the AUC was below 0.70, the sensitivity values were low.

In the ROC analysis for hospital mortality according to the 24 h and 48 h LCs of the patients, the AUC for the 24 h LC was found to be statistically significant (*p* = 0.047); however, the sensitivity value was low because the AUC was below 0.70, as shown in Table 4.

In the ROC analysis performed for hospital mortality according to the patients’ ABE, the AUCs were not statistically significant.

## 4. Discussion

EDs are typically the first point of contact for sepsis patients, where the initial diagnosis is made and treatment is initiated. Utilizing routinely measured biomarkers in blood samples obtained in the emergency setting may assist clinicians in assessing patient prognosis and mortality, thereby guiding therapeutic decisions. The aim of this study was to evaluate the utility of serum lactate, albumin, and BE levels—while controlling for factors that may influence these parameters—in predicting prognosis and mortality in patients diagnosed with sepsis.

The ratio of male patients was 60.4% in our study. In the studies by Ranniko et al. and Morr et al., 53% of the 497 patients and 54% of the 110 patients included in the study were male [14,15]. This rate was consistent with the literature. The mean age of the patients was 67.64 ± 15 years. In the study by Ozaydin et al., the mean age of the patients was 74 years, and in the study by Park et al., it was stated as 54 years [16,17]. The genders, ages, and comorbidities of the patients included in studies vary according to the number of patients and the patient profiles of the hospitals and regions where the studies were conducted.

In our study, 99.1% of the 217 patients had comorbidities. The most common comorbidities were malignancy (56.2%), hypertension (40.1%), and coronary artery disease (51%). Two patients (0.7%) had no known comorbidities. In the study by Javed et al., the most common comorbidities were diabetes mellitus (37%), malignancy (21%), and COPD (20%) [18]. Our hospital is a university hospital with an oncology department. Our ED is visited by a significant number of patients receiving active chemotherapy or with end-stage cancer. This is why cancer patients constituted the majority in our study.

In our study, pulmonary sources were found to be the most common sources of infection, with a proportion of 61.8%. In studies by Javed et al. and Park et al., the most common focus of infection was also found to be pulmonary, at 49% and 62% [17,18], respectively, like in our study.

The 0 and 48 h lactate values did not affect hospital mortality, but it was determined that a high 24th hour lactate value increased hospital mortality. When the lactate values were measured at the initial timepoint and after 6 h, as in the study of Javed et al., lactate values were found to be higher in those who died within 24 h compared to those who survived (*p* < 0.001) [18]. In the study by Ozaydin et al., initial lactate was examined and 28 day mortality was found to be high in those with high lactate (*p* = 0.007) [16]. Our 24 h lactate values increased hospital mortality, similar to the results found in the literature.

It was determined that, in our study, low albumin values at 0, 24, and 48 h increased mortality. In the study by Frenkel et al., serum albumin levels on admission were not associated with in-hospital mortality, but one week after admission, serum albumin levels were significantly associated with the risk of death in patients with sepsis (*p* < 0.001) [19]. In the study by Seo et al., it was found that hypoalbuminemia and low BE increased 28 day mortality (*p* < 0.001) [6]. In the study by Lichtenauer et al., it was determined that high initial lactate values (*p* < 0.001) and low initial albumin values (*p* = 0.01) increased mortality [10]. Low albumin values increased mortality similar to the literature results in our study.

In the study by Yuan et al., it was found that both too low (−41.00 mEq/L to −2.5 mEq/L) and too high BE levels (1.9 mEq/L to 55.5 mEq/L) were associated with a higher risk of 28-day death for sepsis patients [20]. Contrary to the literature, our BE values were not found to have an effect on mortality. This may be related to the fact that the majority of our patients had respiratory problems, and to the exclusion of patients with diseases that would affect their basal metabolic status and also albumin and lactate levels, such as chronic kidney disease and chronic liver disease.

In ROC analyses for lactate, albumin, and BE, no significant AUC was found for BE. Since the AUC values determined for lactate and albumin are below 0.70, their discrimination is low. In the study by Lichtenauer et al., the AUC for lactate was 0.804 and the AUC for albumin was 0.755 [10]. This indicates that they have a high distinctiveness in predicting mortality. However, factors affecting lactate and albumin values were not excluded in this study.

In our study, a statistically significant difference was found when mortality rates were analyzed according to 24 h LC in sepsis patients (*p* = 0.037). Accordingly, as LC decreased at the 24th hour, hospital mortality increased. In the study by Lee et al., mortality was examined according to 6 h LC and it was found that a decrease in LC increased mortality (*p* = 0.001) [4]. In the study by Marty et al., mortality was examined according to 6, 12, and 24 h LCs, and it was found that only a decrease in 24 h LC increased mortality [21]. This is a result similar to that in our study. In ROC analyses for LC, the AUC for 24 h LC is significant, but its discrimination is low because the AUC is below 0.70. Marty et al. found the AUC for 24 h LC to be 0.79, and the threshold value for LC was determined to be −2.1% [21]. In our study, the threshold value could not be determined because the AUC was below 0.70.

In our study, no effect on mortality was determined by ABE values. Therefore, the AUCs were not statistically significant in the ROC analyses for the ABE. The ABE is also an indicator of the kidney function and volume status of patients [8]. In the study by Liu et al., ABE demonstrated a higher AUC (0.580, 95% CI 0.569–0.590) compared to BE (AUC: 0.421, 95% CI 0.410–0.433) and lactate (AUC = 0.444, 95% CI 0.433–0.456), with statistically significant differences (*p* < 0.001). The AUC for the combined model (ABE + SOFA) was significantly higher than the SOFA model alone (for 30 day ICU all-cause mortality, *p* < 0.001) [22]. The sample size of this study (17,099 patients were included) was considerably larger than that of our study. In our study, patients with chronic kidney disease were excluded, and the number of patients receiving vasopressors was small. The fact that our ABE values did not affect mortality may be related to these conditions.

We found that 32.3% (*n* = 70) of the patients died in the hospital. In the study by Freund et al., hospital mortality was 35% in patients with a SOFA score of 2 or higher [23]. This is similar to our mortality rate.

## 5. Conclusions

In our study, it was determined that low 0, 24, and 48 h albumin values, a high 24th hour lactate value, and a decrease in the 24 h LC in sepsis patients increased hospital mortality. On the other hand, BE and ABE values did not affect hospital mortality. The sensitivity of the values affecting mortality was low; therefore, significant results could not be obtained when they were evaluated individually or together. The reason our BE, lactate and albumin values were different from those in the literature may be that all parameters that would affect these values were excluded from the study in our exclusion criteria and because of the comorbidities of our patient population.

Further multicenter studies are needed to evaluate the usefulness of combined biomarkers in predicting morbidity and mortality in sepsis.

In terms of limitations, our study was a single-center study. The majority of our patient profile consists of patients with serious comorbidities such as malignancy and frequent hospital admissions. Therefore, regardless of sepsis, the mortality rates of our patients are high due to their disease and circumstances. We also consider that the lack of statistical significance in our analyses involving patients with septic shock is due to the very limited number of cases in this subgroup.

## Figures and Tables

**Table 1:** SOFA score.

	Score				
**System**	0	1	2	3	4
**Respiration**					
PaO2/FIO2, mmHg	≥400 (53.3)	<400 (53.3)	<300 (40)	<200 (26.7) with respiratory support	<100 (13.3) with respiratory support
**Coagulation**					
Platelets, ×10^3^/μL	≥150	<150	<100	<50	<20
**Liver**					
Bilirubin, mg/dL (μmol/L)	<1.2 (20)	1.2–1.9 (20–32)	2.0–5.9 (33–101)	6.0–11.9 (102–204)	>12.0 (204)
**Cardiovascular**	MAP ≥ 70 mmHg	MAP < 70 mmHg	Dopamine < 5 or any dose dobutamine *	Dopamine 5.1–15 or epinephrine ≤ 0.1 or norepinephrine ≤ 0.1 *	Dopamine > 15 or epinephrine > 0.1 or norepinephrine > 0.1 *
**Central Nervus System**					
Glasgow Coma Scale score **	15	13–14	10–12	6–9	<6
**Renal**					
Creatine, mg/dL (μmol/L)	<1.2 (110)	1.2–1.9 (110–170)	2.0–3.4 (171–299)	3.5–4.9 (300–440)	>5.0 (440)
Urine output, mL/d				<500	<200

Abbreviations: FIO2, fraction of inspired oxygen; MAP, mean arterial pressure; PaO2, partial pressure of oxygen. * Catecholamine doses are given as μg/kg/min for at least 1 h. ** Glasgow Coma Scale scores range from 3 to 15; higher score indicates better neurological function.

**Table 2:** Characteristics of the patients.

Characteristics	*n* = 217
Age yr	67.5 ± 16.5
Sex no. (%)	
Male	131 (60.4)
Female	86 (39.6)
Comorbidities no. (%)	
No disease	2 (0.9)
Malignite	122 (56.2)
Hypertension	87 (40.1)
Coronary Artery Disease	51 (23.5)
Asthma/COPD *	46 (21.7)
Diabetes Mellitus	40 (18.4)
Konjestif Kalp Yetmezliği	31 (14.3)
Alzheimer’s/Parkinson’s/Demans	31 (14.3)
Arrhythmia	24 (11.1)
Cerebrovascular Disease	23 (10.7)
Benign Prostatic Hypertrophy	14 (6.5)
Rheumatological Disease	13 (6)
Hypothyroidism/Hyperthyroidism	10 (4.6)
Epilepsy	8 (3.7)
Others **	48 (22.1)
Sistolic Blood Pressure mmHg	106 ± 44
Mean Arterial Pressure mmHg	79 ± 28
SOFA Scores no. (%)	
2–6	170 (78.3)
7–9	37 (17.1)
10–14	10 (4.6)
Site of Infections no. (%)	
Pulmonary	134 (61.8)
Urinary	36 (16.6)
Bacteremia	25 (11.5)
Intra-abdominal	13 (6)
Soft Tissue	8 (3.7)
Central Nervous System	1 (0.5)

Abbreviations: * COPD: chronic obstructive pulmonary disease. ** adrenal insufficiency, aortic valve replacement, hydrocephalus, cystic fibrosis, mitral valve replacement, osteoporosis, peripheral artery disease, polycythemia vera, pulmonary hypertension, cerebral palsy.

**Table 3:** Lactate, LC, BE, ABE, and albumin values.

	Mean (Std. Deviation)
0 h lactate *	2.40 (1.9)
0 h albumin *	3 (0.7)
0 h BE	−1.47 (6.57)
24 h lactate *	1.7 (1.3)
24 h albumin	2.65 (0.53)
24 h BE	−1.44 (6.04)
48 h lactate *	1.5 (1)
48 h albumin	2.57 (0.51)
48 h BE	−1.3 (6.1)
24 h LC *	26.7 (54.5)
48 h LC *	24 (50)
0 h ABE	1.39 (6.25)
24 h ABE *	0.2 (7.7)
48 h ABE	0.72 (5.56)

* The median (IQR) is given for values that do not show normal distribution. Abbreviations: BE, base excess; ABE, alactic base excess; LC, lactate clearance.

**Table 4:** Hospital mortality according to lactate, LC, BE, ABE, and albumin values.

	Sepsis (*n* = 196)		Septic Shock (*n* = 21)	
	Survival	Nonsurvival	Survival	Nonsurvival
0 h lactate	2.1 (1.5–3.1)	2.65 (1.6–4.5)	2.92 (1.13)	4.04 (1.98)
*p* value	0.056 *		0.14 †	
0 h albumin	3.07 (0.49)	2.68 (0.63)	2.81 (0.57)	2.65 (0.69)
*p* value	0.000 †		0.56 †	
0 h BE	−1.43 (6.1)	−1.01 (7.48)	−1.94 (7.46)	−5.7 (4.95)
*p* value	0.576 †		0.156 †	
24 h lactate	1.5 (1.2–2.1)	2 (1.5–3)	2.2 (1.5–2.9)	2.6 (2.15–4.5)
*p* value	0.001 *		0.268 *	
24 h albumin	2.76 (0.48)	2.49 (0.61)	2.51 (0.45)	2.3 (0.35)
*p* value	0.004 †		0.27 †	
24 h BE	−0.94 (5.35)	−1.29 (6.95)	−4.41 (7.01)	−7.45 (5.29)
*p* value	0.279 †		0.138 †	
48 h lactate	1.5 (1.1–2)	1.6 (1.2–3)	2.05 (1.5–2.55)	1.4 (1.2–2.2)
*p* value	0.058 *		0.151 *	
48 h albumin	2.68 (0.48)	2.37 (0.52)	2.49 (0.47)	2.21 (0.57)
*p* value	0.000 †		0.286 †	
48 h BE	−0.4 (5.13)	−2.03 (6.69)	−3.85 (7.39)	−8.13 (9.44)
*p* value	0.075 †		0.305 †	
0 h ABE	1.32 (5.71)	1.69 (6.92)	1.31 (7.68)	−1.83 (2.79)
*p* value	0.696 †		0.285 †	
24 h ABE	0.93 (5.24)	0.94 (6.49)	−1.22 (7.63)	−4.73 (3.94)
*p* value	0.990 †		0.247 †	
48-h ABE	1.23 (5.11)	0.37 (6.24)	−1.21 (6.54)	−3.07 (5.12)
*p* value	0.330 †		0.553 †	
24 h LC	32.04 (50)	18.75 (56.67)	25.53 (50.37)	27.62 (78.74)
*p* value	0.037 *		0.664 *	
48 h LC	24.57 (54.27)	23.08 (48.05)	21.35 (57.19)	37.88 (142.86)
*p* value	0.430 *		0.640 *	

Abbreviations: * Mann–Whitney Test, median (IQR). † T-Test, mean (std. deviation). BE, base excess; ABE, alactic base excess; LC, lactate clearance.

**Table 5:** Mortality according to SOFA score.

SOFA Score	Sepsis (N = 196) *n*(%)		Septic Shock (N = 21) *n*(%)	
	Survival	Nonsurvival	Survival	Nonsurvival
2–6	115 (71.9)	45 (28.1)	5 (50)	5 (50)
7–9	16 (55.2)	13 (44.8)	7 (87.5)	1 (12.5)
10–14	3 (43.3)	4 (56.7)	1 (33.3)	2 (66.7)
*p* value	0.045		0.188	

**Table 6:** Models for hospital mortality.

	Model-1		Model-2		Model-3	
	AUC	OR	AUC	OR	AUC	OR
0 h lactate	0.517	1.143	-	-	-	1.116
*p* value	0.707	0.038	-	-		0.103
0 h albumin	-	-	0.604	0.267	-	0.281
*p* value	-	-	0.020	0.000		0.103
0 h lactate + albumin	-	-	-	-	0.604	-
*p* value	-	-	-	-	0.019	-
24 h lactate	0.570	1.635	-	-	-	1.580
*p* value	0.125	0.001	-	-		0.001
24 h albumin	-	-	0.540	0.366	-	0.409
*p* value	-	-	0.373	0.002		0.008
24 h lactate + albumin	-	-	-	-	0.580	-
*p* value	-	-	-	-	0.078	-
48 h lactate	0.588	1.610	-	-	-	1.516
*p* value	0.060	0.001	-	-		0.005
48 h albumin	-	-	0.540	0.270	-	0.318
*p* value	-	-	0.395	0.000		0.002
48 h lactate + albumin	-	-	-	-	0.617	-
*p* value	-	-	-	-	0.013	-

**Table 7:** ROC analysis of hospital mortality for BE, ABE, and LC.

	Hospital Mortality	
	AUC	*p* Value
0 h BE	0.500	0.995
24 h BE	0.479	0.639
48 h BE	0.426	0.095
0 h ABE	0.510	0.837
24 h ABE	0.495	0.915
48 h ABE	0.506	0.674
24 h LC	0.412	0.047
48 h LC	0.475	0.580

*Abbreviations: BE, base excess; ABE, alactic base excess; LC, lactate clearance.*

## Data Availability

The data presented in this study are openly available in bioRxiv at doi: https://doi.org/10.1101/2025.03.23.25324310.

## Abbreviations

The following abbreviations are used in this manuscript:

ABE	Alactic base excess
AUC	Area under the curve
BE	Base excess
COPD	Chronic obstructive pulmonary disease
ED	Emergency department
FIO2	Fraction of inspired oxygen
HCC	Hepatocellular carcinoma
ICU	Intensive care unit
LC	Lactate clearance
MAP	Mean arterial pressure
*n*	Number
OR	Odds ratio
PaO2	Partial pressure of oxygen
ROC	Receiver operating characteristic
SOFA	Sepsis-related (sequential) organ failure assessment
Std	Standard
